# Vortex ring mixing in the left ventricle of the human heart

**DOI:** 10.1186/1532-429X-15-S1-E27

**Published:** 2013-01-30

**Authors:** Johannes Toger, Mikael Kanski, Marcus Carlsson, Sándor J Kovács, Gustaf Söderlind, Hakan Arheden, Einar Heiberg

**Affiliations:** 1 Clinical Physiology, Lund University, Skane University Hospital, Lund, Sweden; 2 Numerical Analysis, Centre for Mathematical Sciences, Lund University, Lund, Sweden; 3 Cardiovascular Biophysics Laboratory, Department of Medicine, Cardiovascular Division, Washington University Medical Center, St. Louis, MO, USA

## Background

**Figure 1:**
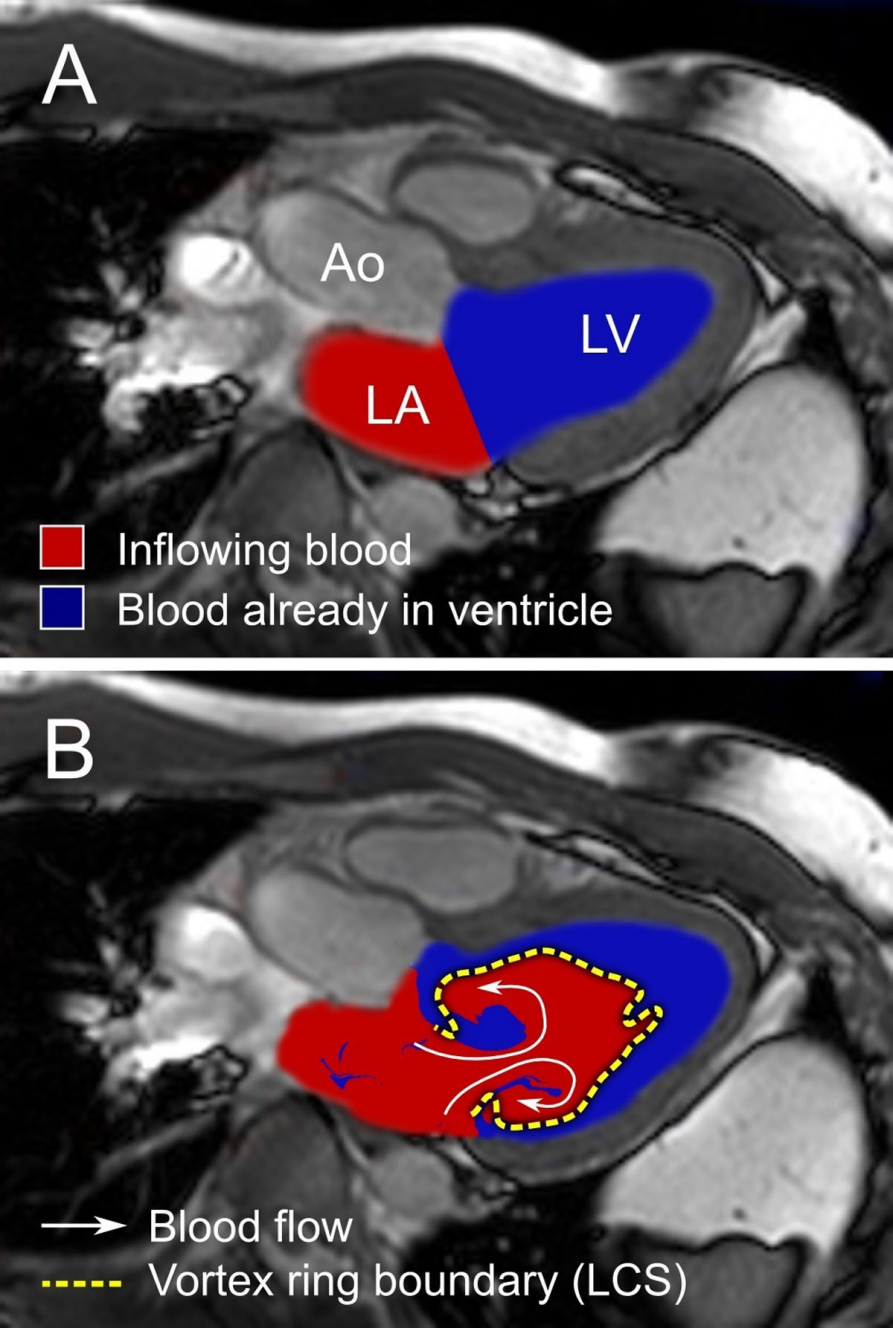
Mixing of blood during vortex ring formation in the left ventricle during rapid filling in a healthy volunteer. Panel A shows the heart before rapid filling, and Panel B shows the heart after rapid filling. The mixing fraction was defined as the volume of the blue voxels inside the vortex ring boundary divided by the total volume of the vortex ring. LA = left atrium, LV = left ventricle, Ao = aorta.

**Figure 2:**
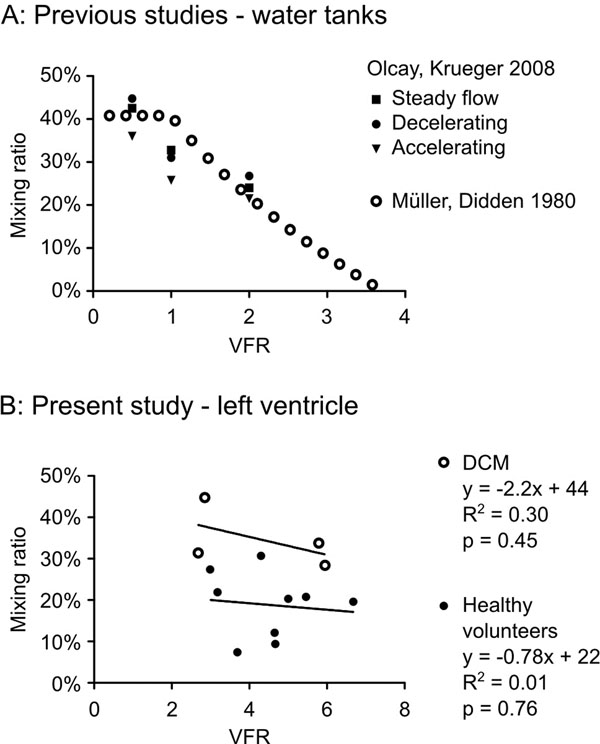
Panel A shows the previous results from experiments in water tanks on the relationship between vortex formation ratio (VFR) and the mixing ratio. The previous studies found that the mixing ratio decreased with increasing VFR. Panel B shows the results from the current study. In contrast to the previous experiments in water tanks, there was no relationship between VFR and the mixing ratio in the left ventricle. Solid lines: linear regression for patients and healthy volunteers respectively.

During rapid filling of the left ventricle, a vortex ring forms downstream from the mitral valve. Previous experiments in water tanks have shown that vortex ring formation is an optimized method for fluid transport. The rotation of the vortex ring leads to mixing of the inflowing blood and blood that was already in the ventricle (Figure 1). In water tanks, the amount of mixing decreases with increasing vortex formation ratio (VFR), a dimensionless parameter relating inflowing volume to the annulus diameter (Figure 2). However, the flow and anatomy of the left ventricle is more complex which may affect this established relationship. Therefore, we aimed to investigate if the relationship between VFR and mixing ratio demonstrated in water tank experiments holds in the human heart.

## Methods

Nine healthy volunteers and four patients with dilated ischemic cardiomyopathy underwent cardiovascular magnetic resonance including 4D PC-MRI. Particle tracing was used to track blood flowing into the left ventricle during rapid filling (Figure 1). Lagrangian Coherent Structures (LCS) were used to define the boundary of the vortex ring. The entrained volume was defined as the volume of blood from the ventricle (Figure 1, colored blue) inside the vortex ring (Figure 1B, yellow line). The mixing fraction was computed as the entrained volume divided by the total vortex ring volume. Differences between patients and healthy volunteers were tested using the Mann-Whitney U test.

VFR was computed as follows: End-systolic volume (ESV) and the volume of the left ventricle at diastasis (diastatic volume, DV) were measured by manual delineations in short-axis cine images. E-wave volume (EWV) was computed as DV-ESV. The diameter of the mitral valve D was measured as the average of the diameters in the three-chamber view and perpendicular to the three-chamber view in the short-axis view. VFR was computed as VFR = 4/π×EWV/D^3^.

## Results

The mixing fraction was significantly higher in the patients compared to the volunteers (35±7% vs. 19±8%, p=0.006). In contrast to previous studies in water tanks, there was no significant correlation between VFR and the mixing ratio in either group (Figure 2B).

## Conclusions

We found a higher mixing ratio in the patients compared to the healthy volunteers. The absence of a significant correlation between VFR and mixing fraction suggests that vortex ring formation in the left ventricle of the human heart is subject to additional complexity and asymmetry compared to experimental studies in water tanks.

## Funding

This study was supported by Swedish Research Council grants VR 621-2005-3129, VR 621-2008-2949 and VR K2009-65X-14599-07-3, National Visualization Program and Knowledge Foundation grant 2009-0080, the Medical Faculty at Lund University, Sweden, the Region of Scania, Sweden and the Swedish Heart-Lung Foundation.

SJK is supported in part by the Alan A. and Edith L. Wolff Charitable Trust, St. Louis, MO, USA, and the Barnes-Jewish Hospital Foundation, St Louis, MO, USA.

